# Switching from Fatty Acid Oxidation to Glycolysis Improves the Outcome of Acute‐On‐Chronic Liver Failure

**DOI:** 10.1002/advs.201902996

**Published:** 2020-02-13

**Authors:** Zujiang Yu, Jingjing Li, Zhigang Ren, Ranran Sun, Yang Zhou, Qi Zhang, Qiongye Wang, Guangying Cui, Juan Li, Ang Li, Zhenfeng Duan, Yuming Xu, Zhichao Wang, Peiyuan Yin, Hailong Piao, Jun Lv, Xiaorui Liu, Yanfang Wang, Ming Fang, Zhengping Zhuang, Guowang Xu, Quancheng Kan

**Affiliations:** ^1^ Department of Infectious Disease The First Affiliated Hospital of Zhengzhou University Zhengzhou 450052 China; ^2^ CAS Key Laboratory of Separation Science for Analytical Chemistry Dalian Institute of Chemical Physics Chinese Academy of Sciences Dalian 116023 China; ^3^ University of Chinese Academy of Sciences Beijing 100049 China; ^4^ Neuro‐Oncology Branch Center for Cancer Research National Cancer Institute National Institutes of Health Bethesda MD 20892 USA; ^5^ Department of Hepatobiliary and Pancreatic Surgery the First Affiliated Hospital School of Medicine Zhejiang University Hangzhou 310003 China; ^6^ Sarcoma Biology Laboratory Department of Orthopaedic Surgery Massachusetts General Hospital and Harvard Medical School Boston MA 02215 USA; ^7^ Department of Pharmacy The First Affiliated Hospital of Zhengzhou University Zhengzhou 450052 China; ^8^ Scientific Research Center for Translational Medicine Dalian Institute of Chemical Physics Chinese Academy of Sciences Dalian 116023 China; ^9^ Ming Fang MD Inc. Walnut Creek CA 94596 USA; ^10^ Surgical Neurology Branch National Institute of Neurological Disorders and Stroke National Institutes of Health Bethesda MD 20892 USA

**Keywords:** acute‐on‐chronic liver failure, hepatocytes, hypoxia, metabolic reprogramming

## Abstract

Acute‐on‐chronic liver failure (ACLF) has a high mortality rate. Metabolic reprogramming is an important mechanism for cell survival. Herein, the metabolic patterns of ACLF patients are analyzed. An in vitro model of ACLF is established using Chang liver cells under hyperammonemia and hypoxia. A randomized clinical trial (ChiCTR‐OPC‐15006839) is performed with patients receiving *L*‐ornithine and *L*‐aspartate (LOLA) daily intravenously (LOLA group) and trimetazidine (TMZ) tid orally (TMZ group) based on conventional treatment (control group). The primary end point is 90‐day overall survival, and overall survival is the secondary end point. By analyzing metabolic profiles in liver tissue samples from hepatitis B virus (HBV)‐related ACLF patients and the controls, the metabolic characteristics of HBV‐related ACLF patients are identified: inhibited glycolysis, tricarboxylic acid cycle and urea cycle, and enhanced fatty acid oxidation (FAO) and glutamine anaplerosis. These effects are mainly attributed to hyperammonemia and hypoxia. Further in vitro study reveals that switching from FAO to glycolysis could improve hepatocyte survival in the hyperammonemic and hypoxic microenvironment. Importantly, this randomized clinical trial confirms that inhibiting FAO using TMZ improves the prognosis of patients with HBV‐related ACLF. In conclusion, this study provides a practical strategy for targeting metabolic reprogramming using TMZ to improve the survival of patients with HBV‐related ACLF.

## Graphical abstract

Metabolic characteristics are identified in patients with acute‐on‐chronic liver failure: inhibited glycolysis, tricarboxylic acid cycle (TAC) and urea cycle, and enhanced fatty acid oxidation (FAO) and glutamine anaplerosis. These effects are mainly attributed to hyperammonemia and hypoxia. Trimetazidine that is used to inhibit FAO could enhance glycolysis, TAC and urea cycle, but inhibit glutamine anaplerosis, thereby improving patients' survival.

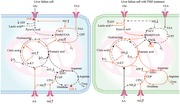

## 1 Introduction

Acute‐on‐chronic liver failure (ACLF) is characterized by acute decompensation of liver function, organ failure(s), and high mortality in patients with chronic liver disease within 4 weeks.^1^ ACLF can develop in patients with any chronic liver disease, including chronic hepatitis B virus (HBV), chronic hepatitis C virus (HCV), alcohol associated liver disease (AALD), and nonalcoholic fatty liver disease (NAFLD). The main cause of ACLF in Asia is chronic HBV infection.^2^ Even with the development of therapeutic strategies, such as antiviral treatment, artificial liver support therapy, and so on, the mortality rate of patients with HBV‐related ACLF just reduced to 50% from 63–72.3%.^3^ Liver transplantation remains the only effective therapy, but a high mortality rate of patients on the waiting list is mainly caused by the rapid disease progression and the lack of donors.^4^ Thus, the aim of this study was to identify an effective therapeutic strategy for patients with ACLF.

Metabolic reprogramming is a common phenomenon and an important mechanism for cells to survive and cope with microenvironment changes, and this phenomenon is widely recognized to occur in tumor cells^5^ and immune cells.^6^ With regard to cancer cells, the most famous metabolic reprogramming is the Warburg effect: limiting their energy metabolism largely to glycolysis even in the presence of oxygen by reprogramming glucose metabolism.^5^ Enhanced glutamine catabolism has been detected in cancer cells.^7^ These metabolic changes provide abundant nutrients and nitrogen atoms,^8^ which are required for cancer cell growth. Similar to the Warburg effect in tumors, the activation of macrophages and dendritic cells (DCs) also involves metabolic switching from oxidative phosphorylation toward glycolysis. In addition, α‐ketoglutarate (α‐KG) that is produced during glutaminolysis is vital for alternatively M2 activated macrophages, which indicates that glutamine metabolism tailors the immune responses of macrophages through metabolic and epigenetic reprogramming.^9^ During ACLF, the hepatocytes must survive in a microenvironment undergoing marked changes. Thus, studying the metabolic reprogramming of the hepatocytes in ACLF may provide a new therapeutic strategy for patients with ACLF.

In this study, we identified the metabolic characteristics of patients with HBV‐related ACLF: inhibited glycolysis, tricarboxylic acid (TCA) cycle and urea cycle, and enhanced fatty acid oxidation (FAO) and glutamine anaplerosis. These effects were mainly attributed to hyperammonemia and hypoxia. Further in vitro study found that switching from FAO to glycolysis could improve hepatocyte survival in the hyperammonemic and hypoxic microenvironment. Importantly, we confirmed, in a randomized clinical trial, that inhibiting the FAO using trimetazidine (TMZ) improved the prognosis for patients with HBV‐related ACLF.

## 2 Results and Discussion

### 2.1 Metabolic Profiles in the Liver Tissue from Patients with ACLF

**Figure 1:**
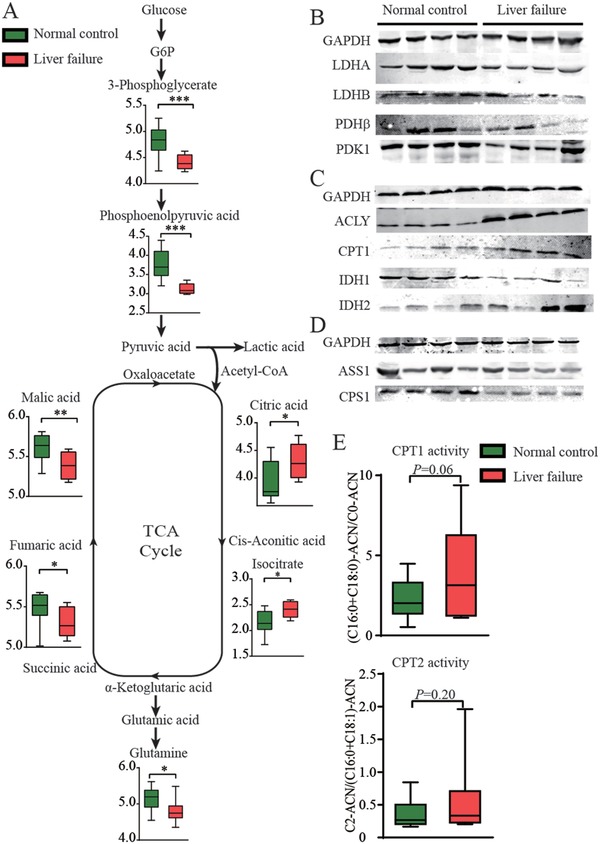
Metabolic profiles in the liver tissue from patients with ACLF. Metabolomics analyses of the liver tissue from 9 patients with ACLF and 15 patients with hepatic hemangioma (normal controls) were performed using GC‐MS and LC‐MS. A) Metabolic changes during glycolysis, oxidative phosphorylation and glutamine anaplerosis in the ACLF liver samples compared with the normal control livers. B–D) Expression of proteins in glycolysis (LDHA and LDHB), oxidative phosphorylation (PDHβ, PDK1), FAO (ACLY and CPT1), glutamine anaplerosis (IDH1 and IDH2) and the urea cycle (ASS1 and CPS1) in the ACLF liver samples compared with the normal control liver samples. E) CPT1 and CPT2 activities were evaluated in the ACLF livers samples compared with the normal control livers samples. The metabolite data were converted to base‐10 logs, and compared using Student's *t‐*tests, *p < 0.05, **p < 0.01.

First, we analyzed the metabolic patterns in liver tissues from 9 patients with HBV‐related ACLF and 15 patients with hepatic hemangioma (normal controls). Compared with the normal controls, the liver tissue from patients with ACLF showed an inhibition of both glycolysis and oxidative phosphorylation, as evidenced by decreased levels of glycerate‐3‐phosphate, phosphoenolpyruvate, fumarate, and malate (**Figure** **1**A). The levels of citrate and isocitrate were increased, while the glutamine level was decreased in the liver tissue from patients with ACLF, suggesting enhanced glutamine anaplerosis (Figure 1A). Consistent with these results, the expressions of enzymes involved in glycolysis and the TCA cycle were inhibited, which could be observed from lactate dehydrogenase A (LDHA), lactate dehydrogenase B (LDHB), pyruvate dehydrogenase (PDHβ), pyruvate dehydrogenase kinase 1 (PDK1) (Figure 1B), and isocitrate dehydrogenase 1 (IDH1) (Figure 1C). The expression of isocitrate dehydrogenase 2 (IDH2) was slightly enhanced, coordinating with the increased glutamine anaplerosis (Figure 1A). Moreover, the expressions of adenosine triphosphate (ATP)‐citrate lyase (ACLY) and carnitine palmitoyltransferase 1 (CPT1) were up‐regulated, and CPT1 activity slightly increased (Figure 1C,E), suggesting enhanced fatty acid metabolic activity, including biosynthesis and oxidation. Additionally, the urea cycle was attenuated in the liver tissue from patients with ACLF, represented by the low expression of the main rate‐limiting enzymes argininosuccinate synthetase 1 (ASS1) and carbamoyl phosphate synthetase 1 (CPS1; Figure 1D). These results shed some light on the metabolic characteristics of dysfunctional hepatocytes in patients with ACLF: inhibited glycolysis, TCA cycle and urea cycle, but enhanced FAO and glutamine anaplerosis.

### 2.2 Hyperammonemia, in the Context of Hypoxia, Contributes to the Metabolic Pattern Alterations in the ACLF Liver Tissue

To understand the mechanism of metabolic reprogramming in ACLF, we established an in vitro model mimicking the microenvironment of the hepatocytes in patients with ACLF. Currently, hypoxia (including hypoxia‐associated oxidative stress and ATP shortage) is considered as the main factor that promotes liver failure (LF) progression.^10^ Thus, we chose the Chang liver cells, which bear normal hepatic function and are often used to investigate liver diseases^11^ to test the metabolic status of the hepatocytes under hypoxic conditions. We identified that 96 metabolites and 57 metabolic pathways were altered in cells exposed to hypoxia (Figure S1A,B, Supporting Information). Similar metabolic alterations in the TCA cycle, urea cycle, and FAO were observed in the hepatocytes exposed to hypoxia. However, the enhanced glycolysis and decreased glutamine anaplerosis, which differed from the metabolic profiles of tissues from ACLF patients (Figure S1C, Supporting Information) were also observed. These data indicate that hypoxia alone could not induce the metabolic changes that completely match the metabolic characteristics in patients with ACLF.

**Figure 2:**
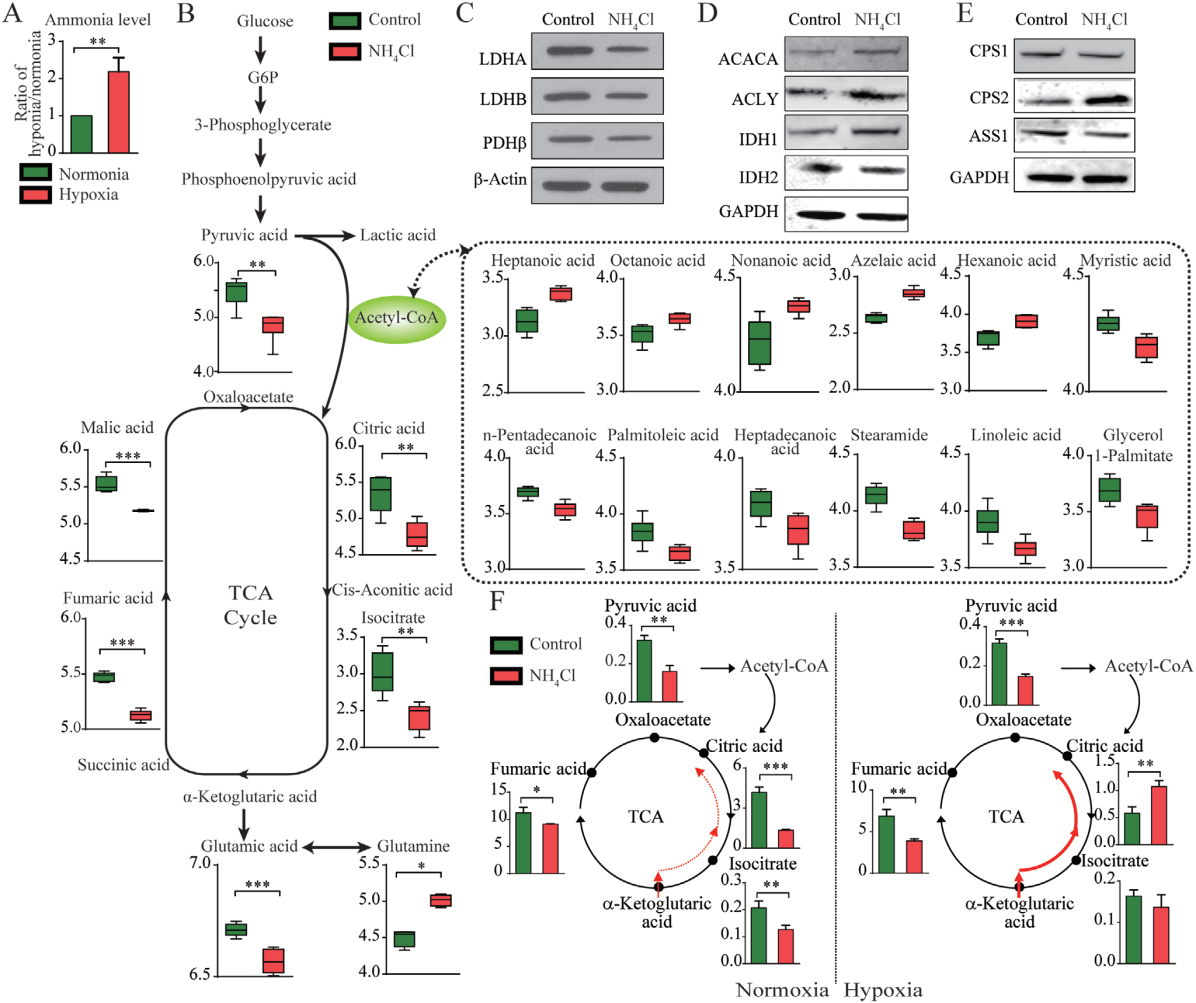
Metabolic status of the Chang liver cells exposed to hyperammonemia and hypoxia. A) Hypoxia increased ammonia accumulation in the Chang liver cells. B) Metabolomic analyses of the Chang liver cells exposed to hyperammonemia were performed using GC–MS. Metabolic changes in glycolysis, oxidative phosphorylation, FAO, and glutamine anaplerosis in the Chang liver cells exposed to hyperammonemia. C–E) Expressions of proteins during glycolysis (LDHA and LDHB), oxidative phosphorylation (PDHβ), FAO (ACLY and CPT1), glutamine anaplerosis (IDH1 and IDH2), and the urea cycle (ASS1, CPS1, and CPS2) in the Chang liver cells exposed to hyperammonemia. F) Metabolic changes during glycolysis, oxidative phosphorylation, and glutamine anaplerosis in the Chang liver cells exposed to hyperammonemia and hypoxia. The data were compared using Student's *t‐*tests, *p < 0.05, **p < 0.01, ***p < 0.001.

We found that ammonia accumulation, another crucial physiological and pathological event in ACLF,^12^ was induced by hypoxia in vitro (**Figure** **2**A). Next, the metabolic profiles of the Chang liver cells exposed to hyperammonemia were evaluated (Figure S2A,B, Supporting Information). Glycolysis, the TCA cycle, the urea cycle (presented by the ratio of Citrulline/Ornithine [Cit/Orn] and the levels of CPS1 and ASS1), and glutamine anaplerosis were inhibited, but FAO was enhanced (Figure 2B–E and Figure S2E, Supporting Information). To further determine the metabolic changes in response to ammonia exposure, a stable isotope labeling technique was used. When fully ^13^C‐labelled glucose was taken up by the Chang liver cells, the M+3 isotopomer of pyruvate and the M+2 isotopomers of TCA cycle metabolites were reduced in cells exposed to the ammonia (Figure S2C, Supporting Information). Moreover, by fully labeling the carbon of glutamine, we found inhibited glutamine anaplerosis in the hepatocytes exposed to ammonia (Figure S2D, Supporting Information). These results confirmed that ammonia inhibited glycolysis, TCA cycle, urea cycle, and glutamine anaplerosis, but enhanced fatty acid metabolism in the Chang liver cells. These metabolic changes, except the glutamine anaplerosis, were more similar to those observed in patients with ACLF. We found that the combination of hyperammonemia and hypoxia inhibited glycolysis and glutamine anaplerosis (Figure 2F). Thus, we speculated that hyperammonemia, in the context of hypoxia, mainly contributed to the metabolic pattern alterations in the ACLF liver tissue.

### 2.3 Metabolism‐Based Mechanism for the Survival of the Chang Liver Cells after Exposure to Hyperammonemia and Hypoxia

**Figure 3:**
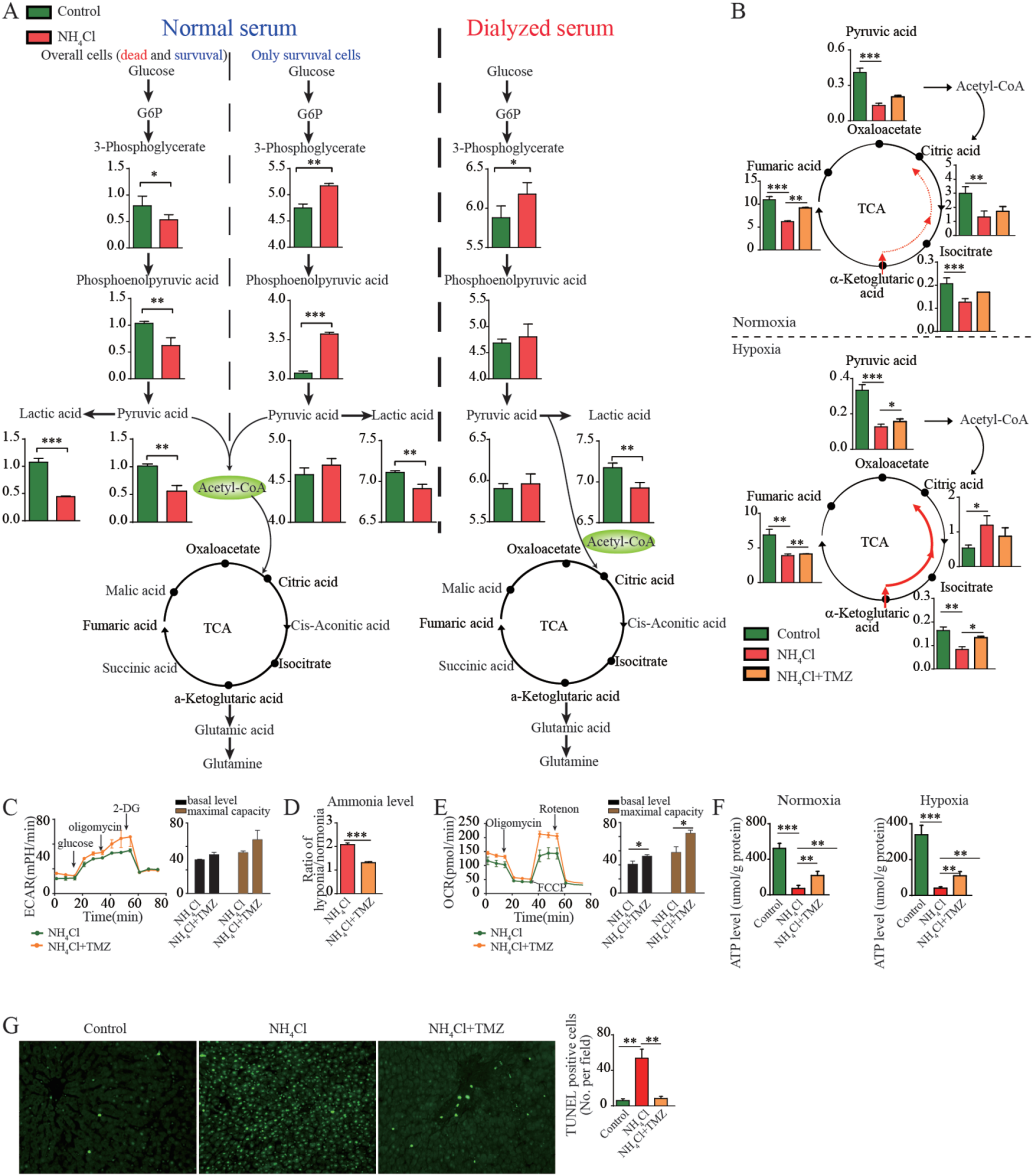
Metabolism‐based mechanisms for the survival of the Chang liver cells after exposure to hyperammonemia and hypoxia. A) Differences in the metabolic patterns between the surviving Chang liver cells and the whole Chang liver cell population (including both surviving and dead cells) after exposure to hyperammonemia (left and middle panels) and the effect of FAO on glycolysis (right). B) Effect of TMZ on glycolysis, as evidenced by metabolite changes and C) ECAR. D) Ammonia levels in the Chang liver cells exposed to hyperammonemia after TMZ administration. Effect of TMZ on oxidative phosphorylation, as shown by E) OCR and F) ATP generation. G) Effect of TMZ on apoptosis evaluated in the liver tissue from rats exposed to NH_4_Cl. The data were compared using Student's *t*‐tests, *p < 0.05, **p < 0.01, ***p < 0.001.

The aim of therapy for patients with ACLF is to best protect the live hepatocytes for cell regeneration. To explore the mechanism of hepatocyte survival during ACLF, we compared the differences in metabolic patterns between the surviving Chang liver cells and the whole population of the Chang liver cells (including both surviving and dead cells) exposed to hyperammonemia. In contrast to the whole cell population, the surviving cells exhibited enhanced glycolysis (**Figure** **3**A, left and middle panel). Enhanced glycolysis is known to be beneficial for tumor cell proliferation and the attenuation of apoptosis by providing glycolytic intermediates for various biosynthetic pathways.^5^, ^13^ Thus, we speculated that enhanced glycolysis might play a similar role in helping the hepatocytes survive under conditions of hypoxia and hyperammonemia.

Hence, hypoxia, hyperammonemia and glycolysis may be the targets for the ACLF treatment. With regards to hypoxia, there are no effective therapies or drugs for ACLF or other types of organ failure. Additionally, hypoxia inhibited glycolysis in the Chang liver cells (Figure 1A,B). Regarding hyperammonemia, the drug *L*‐ornithine and *L*‐aspartate (LOLA) is usually administrated to patients with hepatic encephalopathy because it removes ammonia by increasing urea generation in the urea cycle.^14^ However, our previous study demonstrated that LOLA did not improve cell survival under conditions of hyperammonemia in vitro^15^ or improve patient survival in LF,[qv: 11a] similar to the findings presented in our randomized clinical trial (SMT group vs LOLA group, *p* = 0.989, **Table** **1**).

**Table 1:** Baseline characteristics of the included patients. All values are expressed as means ± standard deviations, medians (with 95% confidence intervals [CI]) or numbers (and percentages). Continuous variables were compared using an analysis of variance (ANOVA) analysis, and categorical variables were compared using chi‐squared tests

Parameters	SMT^c)^ (*n* = 45)	LOLA^c)^ (*n* = 48)	TMZ^c)^ (*n* = 39)	*P* value
Age (years)	48.11 ± 12.095	44.44 ± 10.806	43.92 ± 14.245	0.222
Gender (male)	34 (75.6%)	36 (75.0%)	33 (84.6%)	0.495
Weight [kg]	66.71 ± 10.44	67.04 ± 12.24	66.31 ± 10.78	0.955
RBC count^c)^ (10^3^/mm^3^)	3.65 (3.47–3.84)	3.66 (3.38–3.94)	3.58 (3.27–3.85)	0.839
WBC count^c)^ (10^3^/mm^3^)	8.30 (6.88–9.73)	7.58 (6.39–8.78)	6.52 (5.55–7.49)	0.137
Platelet count (10^3^/mm^3^)	75.20 (63.42–89.98)	83.65 (68.33–98.96)	78.23 (66.94–89.52)	0.639
ALT [U L^−1^]^c)^	394.56 (242.14–546.97)	422.00 (252.90–591.10)	484.41 (288.42–680.40)	0.761
AST [U L^−1^]^c)^	385.22 (269.29–501.16)	341.67 (213.14–470.20)	361.45 (200.68–522.81)	0.893
Creatinine [mg dL^−1^]	1.038 (0.70–1.37)	0.71 (0.60–0.82)	0.83 (0.68–0.98)	0.101
Bilirubin [mg dL^−1^]	19.44 (16.51–22.37)	21.41 (18.55–24.28)	18.01 (15.06–20.96)	0.254
INR^c)^	2.24 ± 0.56	2.40 ± 0.62	2.28 ± 0.78	0.467
Serum sodium [mmol L^−1^]	136.24 ± 4.68	135.66 ± 6.66	136.58 ± 4.11	0.721
HBV DNA [IU L^−1^]				
<500^a)^	27 (60%)	27 (56.3%)	22 (56.4%)	0.921
≥500^b)^	5.56 ± 1.96	5.96 ± 1.35	5.86 ± 1.84	0.285
Ammonia [µmol L^−1^]	96.30 (79.52–113.08)	88.20 (61.56–114.85)	114.20 (66.84–161. 56)	0.491
MELD score^c)^	24.04 ± 6.82	23.02 ± 4.48	22.89 ± 5.65	0.587
Survival	16(35.6%)	17(35.4%)	25 (64.1%)	0.010

^a)^ The number of patients with HBV DNA virus load <500 IU L^−1^

^b)^ HBV DNA virus load shown by mean ± standard deviation (lg10) in patients with HBV DNA virus load ≥ 500 IU L^−1^

^c)^ ALT, alanine transaminase; AST, aspartate transaminase; INR, international normalized ratio; MELD, model for end‐stage liver disease; LOLA, *L*‐ornithine and *L*‐aspartate; RBC, red blood cell; SMT, supportive medical treatment; TMZ, trimetazadine; WBC, white blood cell.

Next, we focused on a method way to enhance glycolysis. Our earlier study found that directly enhancing glycolysis by fructose diphosphate (FDP) failed to improve the prognosis in patients with ACLF (Table S3, Supporting Information), which was inconsistent with the results we expected. To study metabolic reprogramming in ACLF in more detail, we focused on the enhancement of FAO, because FAO is considered as the main energy source for cells, in addition to glycolysis and oxidative phosphorylation. Using dialyzed serum (in which the fatty acids were removed), we found that the glycolysis was increased in the hepatocytes exposed to hyperammonemia (Figure 3A, right panel). Furthermore, the Chang liver cells were cultured in either normal serum or dialyzed serum, and palmitic acid was added to observe the effect of FAO on glycolysis. We observed that glycolysis and the TCA cycle were inhibited regardless of whether the cells were cultured in medium with normal or dialyzed serum (Figure S3A–D, Supporting Information). Moreover, the addition of palmitic acid induces lower pyruvate levels in cells exposed to hyperammonemia and hypoxia (Figure S3A,D, Supporting Information). These data indicated that glycolysis was inhibited by FAO in the hepatocytes exposed to hyperammonemia and hypoxia.

Based on our insight into the mechanism of metabolic reprogramming in patients with ACLF, we proposed a novel treatment strategy for patients with ACLF: inhibiting FAO. Fatty acid oxidation is mainly catalyzed by the chain of β‐oxidase in mitochondria, in which 3‐ketoacyl‐CoA thiolase is one of the catalytic oxidase systems of the β‐oxidation chain. Trimetazidine is a specific inhibitor of 3‐ketoacyl‐CoA thiolase that attenuates cardiomyocyte injury by switching the energy metabolism from FAO to glycolysis and has been used for decades,^16^ thus, it is a potential candidate for inhibiting FAO in the hepatocytes. Moreover, TMZ is normally excreted in urine as a prototype and the hepatotoxicity is unknown according to the drug package insert. A previous study proved that TMZ may ameliorate ischemia reperfusion injury in liver resection and does not increase risk of liver damage and LF,^17^ indicating that TMZ is most likely safe for use in patients with ACLF. As expected, we found that TMZ increased glycolysis, as evidenced by increased pyruvate levels and high extracellular acidification rate (ECAR) at maximal capacity, in the Chang liver cells exposed to hyperammonemia (Figure 3B,C), which was accompanied by reduced ammonia levels (Figure 3D). Intriguingly, it also increased the oxygen consumption rate (OCR) at both the basal level and maximal capacity and increased ATP generation, which suggested an enhanced oxidative phosphorylation (Figure 3E,F). Furthermore, TMZ effectively decreased the apoptosis rate in liver tissue from rats exposed to NH_4_Cl (Figure 3G).

### 2.4 Therapeutic Efficacy of Inhibiting FAQ via TMZ in Patients with ACLF

**Figure 4:**
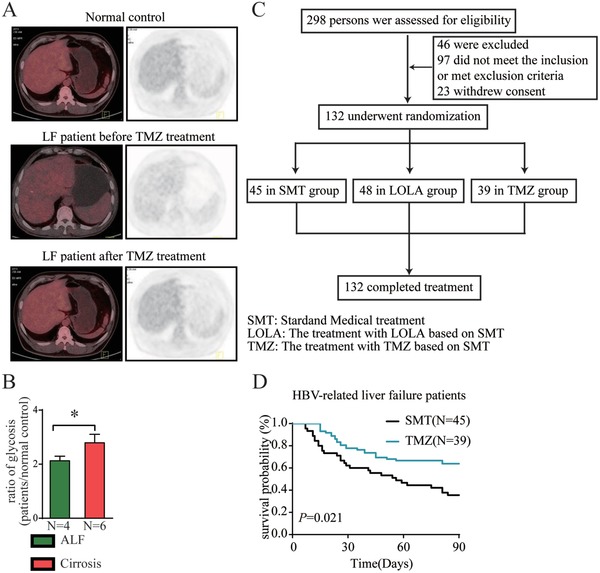
Efficacy of TMZ in patients with ACLF was evaluated. A) Glycometabolism in the liver tissue from normal controls and patients with ACLF before and after TMZ treatment was evaluated via 18F‐FDG uptake and PET‐CT analysis. B) Glycometabolism in liver samples from patients with ACLF and patients with cirrhosis were compared by PET‐CT. The data were compared using Student's t‐tests, *p < 0.05. C) Flow diagram of a randomized clinical trial for patients with ACLF divided into three groups: SMT, LOLA, and TMZ. D) The overall survival (OS) of patients with LF after treatment in the two groups was analyzed by Kaplan–Meier analysis.

Next, we evaluated the efficacy of TMZ in patients with ACLF. First, glycometabolism in the liver tissue from patients with ACLF was assessed by positron emission tomography‐computed tomography (PET‐CT). We found that the liver samples from patients with ACLF showed a low uptake of ^18^F‐fludeoxyglucose (^18^F‐FDG) compared with the liver samples from normal control and patients with cirrhosis (**Figure** **4**A,B). Moreover, the ^18^F‐FDG uptake recovered in patients who were administrated with TMZ (Figure 4A). Thus, a randomized clinical trial was performed (Figure 4C and Table 1). We found that TMZ markedly increased the overall survival (OS) of patients with ACLF using a Kaplan–Meier analysis (Figure 4D). A subgroup analysis revealed that TMZ significantly improved the prognosis of patients with alanine aminotransferase (ALT) or aspartate transaminase (AST) levels less than 400 U L^−1^ (**Tables** **2** and **3**). Because severe ACLF usually leads to low levels of transaminases, these results suggested that TMZ was especially valuable in patients with severe ACLF patients.

**Table 2:** Analysis of the 90‐day mortality stratified by levels of alanine transaminase. All values are expressed as numbers (and percentages). Categorical variables were compared using chi‐squared tests

ALT^a)^ [U L^−1^]	Outcome	SMT^a)^	TMZ^a)^	*P* value
>400	Death	6 (40%)	4 (26.7%)	0.4386
	Survival	9 (60%)	11 (72.3%)	
≤400	Death	23 (76.9%)	10 (25%)	0.0088
	Survival	7 (23.1%)	14 (75%)	

^a)^ ALT, alanine transaminase; SMT, supportive medical treatment; TMZ, trimetazidine.

**Table 3:** Analysis of the 90‐day mortality stratified by levels of aspartate transaminase. All values are expressed as numbers (and percentages). Categorical variables were compared using chi‐squared tests

AST [U L^−1^]^a)^	Outcome	SMT^a)^	TMZ^a)^	*P* value
>400	Death	5 (50.0%)	4 (45.5%)	0.3918
	Survival	7 (50.0%)	6 (54.5%)	
≤400	Death	24 (81.3%)	10 (32.0%)	0.0025
	Survival	9 (18.8%)	19 (68.0%)	

^a)^ AST, aspartate transaminase; SMT, supportive medical treatment; TMZ, trimetazidine.

## 3 Conclusion

In summary, we identified the metabolic profiles in patients with ACLF, which were mainly attributed to hyperammonemia in the context of hypoxia. Moreover, enhanced glycolysis might help the hepatocytes survive under conditions of hyperammonemia and hypoxia. Importantly, glycolysis was inhibited by FAO in hepatocytes exposed to hyperammonemia. Therefore, we proposed a novel strategy that TMZ was proven effective for ACLF in a randomized clinical trial. Currently, TMZ use in patients with ACLF has been approved by the FDA as an orphan drug treatment. This study provides a practical strategy, targeting metabolic reprogramming by switching from FAO to glycolysis with TMZ, to improve the survival of patients with ACLF.

## 4 Experimental Section

##### Randomized Clinical Trial of Acute‐On‐Chronic Liver Failure

The trial was approved by the ethics committee of the First Affiliated Hospital of Zhengzhou University and was registered in the Chinese Clinical Trial Registry (registration number: ChiCTR‐OPC‐15006839). The trial was conducted in accordance with the Declaration of Helsinki and Good Clinical Practice. All patients signed informed consent before enrollment. The inclusion and exclusion criteria are listed in Table S1 (Supporting Information). A computer‐generated randomization table was used for randomization. All included patients received conventional treatment including magnesium isoglycyrrhizinate, reduced glutathione and/or albumin. For the *L*‐ornithine and *L*‐aspartate group, 10 mg of LOLA daily was intravenously injected based on the conventional treatment. For the TMZ group, TMZ (20 mg, three times per day) was administered orally for four weeks based on the conventional treatment.

##### Cell Culture and Reagents

The Chang liver cells were purchased from the Cell Bank of the Chinese Academy of Sciences (Shanghai, China). The cells were cultured in RPMI1640 medium (Solarbio Science & Technology Co., Ltd, Beijing, China) supplemented with 10% foetal bovine serum (FBS; Gibco; Thermo Fisher Scientific Inc., Waltham, MA USA) and 1% penicillin/streptomycin (Beijing Solarbio) and were maintained at 37.0 ± 0.2 °C in a humidified incubator with 5.0% CO_2_. In all experiments, a single combination treatment of 10 × 10^−3^ m NH_4_Cl, 1 × 10^−3^ m LOLA and 1 × 10^−3^ m TMZ was performed unless otherwise indicated for 24 or 48 h.

##### Oxygen Consumption and Extracellular Acidification Assessments

The oxygen consumption rate and extracellular acidification rate were assessed using a Seahorse XFe96 Analyzer (Agilent Technologies, Santa Clara, CA, USA). The Chang liver cells were seeded in 7 or 8 wells of a special 96‐well plate at a density of 20 000 cells per well. Cells were then cultured in medium with chemicals as indicated for 48 h. Glycolytic activity and mitochondrial function were tested using the Seahorse XF Glycolysis Stress Test Kit and the XF Cell Mito Stress Test Kit (Agilent Technologies), respectively, according to the manufacturer's instructions.

##### Western Blot Assay

These experiments were performed as previously described.^18^ The following primary antibodies were obtained from Cell Signaling Technology Inc. (Danvers, MA USA) and were used at a 1:1000 dilution: anti‐adenosine triphosphate‐[ATP‐]citrate lyase (anti‐ACLY), anti‐isocitrate dehydrogenase 1 (anti‐IDH1), anti‐isocitrate dehydrogenase 2 (anti‐IDH2), anti‐carnitine palmitoyltransferase 1 (anti‐CPT1), anti‐carbamoyl phosphate synthetase 1 (anti‐CPS1), anti‐carbamoyl phosphate synthetase 2 (anti‐CPS2), anti‐argininosuccinate synthetase 1 (anti‐ASS1), anti‐pyruvate dehydrogenase kinase 1 (anti‐PDK1), anti‐lactate dehydrogenase A (anti‐LDHA), anti‐lactate dehydrogenase B (anti‐LDHB), anti‐pyruvate dehydrogenase (anti‐PDHβ), anti‐acetyl‐CoA carboxylase alpha (anti‐ACACA), and anti‐glyceraldehyde 3‐phosphate dehydrogenase (anti‐GAPDH). The secondary antibodies were horseradish peroxidase‐conjugated goat anti‐rabbit (1:5000; LI‐OR, c60329‐15) and goat anti‐mouse antibodies (1:15 000; LI‐OR, c60405‐05).

##### Gas Chromatography–Mass Spectrometry (GC–MS) and Liquid Chromatography–Mass Spectrometry (LC–MS) Metabolomics

The Chang liver cells were cultured in 10 cm plates with 3 to 6 replicates per group and treated with chemicals as indicated for 48 h. Cells were harvested with 1 mL of methanol solution after being washed with ice‐cold phosphate buffered saline (PBS) and then were resuspended in water and lysed by sonication. The supernatants were lyophilized. For the tissue samples, 20 mg of tissue was mixed with 1 mL of 80% MeOH solution containing internal standards (IS) (1 µg mL^−1^ succinic acid‐d_4_ and citric acid‐d_4_) and homogenized. The supernatants were lyophilized.

For GC–MS analysis, the lyophilized samples were subjected to oximation and silylation reactions. A QP 2010 GC‐MS plus gas chromatograph‐mass spectrometer (Shimadzu Corp., Kyoto, Japan) coupled with a J&W DB‐5 ms fused‐silica capillary column (Agilent Technologies) was used for metabolic profiling. The pseudo‐targeted method was established as previously reported.^19^ In total, 252 metabolites from cell samples and 290 metabolites from tissue samples were determined for pseudo‐targeted data collection and quantification. The system parameter settings and metabolite identification were similar to those in a previous report.^20^

For LC‐MS analysis, the lyophilized samples were re‐dissolved in acetonitrile/water (v/v 1:4) solvent and then analyzed by ultra‐performance liquid chromatography (UPLC; Waters Corp, Milford, MA, USA) coupled to a tandem linear ion‐trap quadrupole (LTQ) Orbitrap XL mass spectrometer (Thermo Fisher Scientific, Rockville, MD). The metabolites were separated on an ACQUITY HSS T3 column (2.1 mm × 50 mm, 1.7 µm, Waters Corp.). The mobile phases used water containing 0.1% formic acid (A) and acetonitrile (B). The elution gradient started with 5% B for 0.5 min, increased to 40% B at 2 min and then to 100% B at 8 min and remained at 100% B for 2 min. The flow rate was 0.4 mL min^−1^. The data were collected in electrospray ionization‐positive (ESI+) mode. The mass scan range was 80–800 m/z, and the resolution was 15.000. Peak integration was performed using Xcalibur (V2.2, Thermo Fisher Scientific) software based on the theoretical m/z of carnitine molecules.

##### ^13^C‐Labeling Experiments

One million Chang liver cells were seeded in 10 cm plates in triplicate and cultured in complete medium with 11 × 10^−3^ m ^13^C_6_‐glucose or 2 × 10^−3^ m ^13^C_5_‐glutamine for a total of 48 h with or without chemical treatment. Lyophilized samples were prepared as described previously and subjected to oximation and silylation reactions using methoxamine solution and *N*‐methyl‐*N*‐(*tert*‐butyldimethylsilyl) trifluoroacetamide containing 1% *tert*‐butyldimethylchlorosilane, respectively. One microliter of supernatant was injected in splitless mode. Helium (99.9995%) was used as the carrier gas at a speed of 40 cm s^−1^. The oven temperature was held at 100 °C for 3 min, increased to 310 °C at 3.5 °C min^−1^ and held at 310 °C for 5 min. Data were collected in selected ion monitoring (SIM) mode. The parameters used are shown in Table S2 (Supporting Information).

##### Ammonia Detection

The Chang liver cells were seeded in 6‐well plates and treated with the indicated chemicals (10 × 10^−3^ m NH_4_Cl, 1 × 10^−3^ m LOLA, and 1 × 10^−3^ m TMZ) for 48 h with or without hypoxia (1% O_2_). Supernatants were then harvested, and ammonia levels were determined according to the manufacturer's instructions (K‐AMIAR, Megazyme Ltd., Bray, Ireland). Fluorescence was detected using a microplate spectrophotometer (Spectramax i3x, Molecular Devices, San Jose, CA USA) at a wavelength of 340 nm.

##### Establishment and Treatment of the Rat Model

Animal experiments were approved by the Animal Care and Use Committee of Zhengzhou University and all experimental procedures involving animals were strictly followed in accordance with the Guide for the Care and Use of Laboratory Animals. Forty male Sprague Dawley (SD) rats were randomly assigned to five groups (control, NH_4_Cl, and TMZ) with 8 rats in each group. For all groups except the control group, 10 mL kg^−1^ of a 10% NH_4_Cl solution was administrated intragastrically at 8:00 and 20:00 daily. The rats in the TMZ groups were further treated with 2 mg kg^−1^ TMZ, respectively. Rats in the control group were treated with 10 mg kg^−1^ of normal saline. After 30 days, the rats were sacrificed, and blood samples were harvested from the heart for the detection of ammonia, alanine transaminase and aspartate transaminase. The liver tissue was also collected.

##### Terminal Deoxynucleotidyl Transferase dUTP Nick End Labeling (TUNEL) Assays

The liver samples were collected from the treated rats. The apoptotic hepatocytes in 3 µm formaldehyde‐fixed paraffin‐embedded sections were detected using the In Situ Cell Death Detection Kit (Roche, Basel, Switzerland) and fluorescence detection according to the manufacturer's instructions. The positive cells were counted in ten fields at 400x magnification, and five sections of each sample were analyzed.

##### Statistical Analysis

For GC–MS data, the total peak areas of raw data from cell samples were normalized. For tissue samples, the raw data were first normalized to IS and further normalized to tissue weight. For ^13^C‐labelled experiments, raw data were normalized to natural isotopes using self‐programmed software. The metabolite data were converted to base‐10 logs, compared using a Student's *t‐*test and visualized using Multiple Experiment Viewer (tm4.org). Metabolite‐associated pathways were analyzed using MetaboAnalyst 2.0 (Xia Lab at McGill University, Montreal, QC Canada; metaboanalyst.ca). A *P*‐value of less than 0.05 was used to indicate statistical significance. For the clinical data of patients, all values are expressed as means ± standard deviations, medians (with 95% confidence intervals [CIs]), or numbers (%). Continuous variables were compared using analysis of variance (ANOVA) testing, and categorical variables were compared using chi‐squared tests.

## Conflict of Interest

The authors declare no conflict of interest.

## Author Contributions

Z.Y., Q.K., Z.Z., and G.X. conceived the idea and designed the study. J.L., Z.R., R.S., Y.Z., Q.Z., Q.W., G.C., J.L., A.L., Y.X., Z.W., P.Y., H.P., J.L., X.L., and Y.W. performed the experiments and analyzed the data. Z.D. and M.F. contributed to the design and interpretation of the study. Z.Y., Q.Z., G.C., J.L., Z.R., R.S., and G.X. wrote the manuscript. All authors approved the final version of manuscript.

## Supporting information

Supporting Information

Supporting Information
