# Supplementary material for: Switching from Fatty Acid Oxidation to Glycolysis Improves the Outcome of Acute‐On‐Chronic Liver Failure
Source: Adv Sci (Weinh). 2020 Feb 13;7(7):1902996. doi: 10.1002/advs.201902996 (PMC7141014; doi:10.1002/advs.201902996)
Supplement: Supplementary file 1 — Supporting Information [file ADVS-7-1902996-s001.pdf]

Copyright WILEY-VCH Verlag GmbH & Co. KGaA, 69469 Weinheim, Germany, 2020.

ADVANCED SCIENCE Open Access

Supporting Information

for *Adv. Sci.,* DOI: 10.1002/advs.201902996

Switching from Fatty Acid Oxidation to Glycolysis Improves the Outcome of Acute-On-Chronic Liver Failure

Zujiang Yu, Jingjing Li, Zhigang Ren, Ranran Sun, Yang Zhou, Qi Zhang, Qiongye Wang, Guangying Cui, Juan Li, Ang Li, Zhenfeng Duan, Yuming Xu, Zhichao Wang, Peiyuan Yin, Hailong Piao, Jun Lv, Xiaorui Liu, Yanfang Wang, Ming Fang, Zhengping Zhuang,* Guowang Xu,* and Quancheng Kan**

Supplementary Tables and Figures

Title

Switching from fatty acid oxidation to glycolysis improves the outcome of acute-on-chronic liver failure

Authors

Zujiang Yu[1†*], Jingjing Li[1†], Zhigang Ren[1†], Ranran Sun[1†], Yang Zhou[2,8†], Qi Zhang[3,4], Qiongye Wang[1], Guangying Cui[1], Juan Li[1], Ang Li[1], Zhenfeng Duan[5], Yuming Xu[6], Zhichao Wang[2,7,8], Peiyuan Yin[2], Hailong Piao[7], Jun Lv[1], Xiaorui Liu[1], Yanfang Wang[6], Ming Fang[9], Zhengping Zhuang[3,10*], Guowang Xu[2,8*], Quancheng Kan[6*]

Affiliations

[1] Department of Infectious Disease, the First Affiliated Hospital of Zhengzhou University, Zhengzhou 450052, China

[2] CAS Key Laboratory of Separation Science for Analytical Chemistry, Dalian Institute of Chemical Physics, Chinese Academy of Sciences, Dalian 116023, China

[3] Neuro-Oncology Branch, Center for Cancer Research, National Cancer Institute, National Institutes of Health, Bethesda, Maryland, USA

[4] Department of Hepatobiliary and Pancreatic Surgery, the First Affiliated Hospital, School of Medicine, Zhejiang University, Hangzhou 310003, China

[5] Sarcoma Biology Laboratory, Department of Orthopaedic Surgery, Massachusetts General Hospital and Harvard Medical School, Boston, Massachusetts, USA

[6] Department of Pharmacy, the First Affiliated Hospital of Zhengzhou University, Zhengzhou 450052, China

[7] Scientific Research Center for Translational Medicine, Dalian Institute of Chemical Physics, Chinese Academy of Sciences, Dalian 116023, China

[8] University of Chinese Academy of Sciences, Beijing, China

[9] Ming Fang MD Inc., Walnut Creek, California, USA

[10] Surgical Neurology Branch, National Institute of Neurological Disorders and Stroke, National Institutes of Health, Bethesda, Maryland, USA

†These authors contributed equally to this work.

Supplementary Tables

Table S1. The inclusion and exclusion criteria of the randomized controlled trial on liver failure

**Inclusion criteria**	Consecutive patients with a diagnosis of liver failure according to the consensus recommendations of the Asian Pacific Association for the Study of the Liver (APASL) and European Association for the Study of the Liver (EASL) about the acute-on-chronic liver failure based on chronic HBV infection.
	Age between 14 and 75 years old
	Hospitalized patients
	Informed consent of the patients or the nearest relatives
**Exclusion criteria**	Liver failure of pregnancy, HELLP syndrome, or other viral infection
	Liver failure secondary to intrahepatic malignancy
	Patients who allergy to any constituents of trimetazidine
	Parkinson disease, parkinsonian symptoms, tremors, restless leg syndrome, and other related movement disorders
	Significant renal function impairment (creatinine clearance rate less than 30 ml/min)
	Females who are lactating or pregnant or those who plan to become pregnant during the study
	Patients with a diagnosis of myocardial infarct in the past six months
	Other factors that are not suitable for the study of the test

Table S2. Parameters used in ^{13}C-labeling experiments

**Metabolites**	**Retention time (min)**	**m/z**
Pyruvate	8.45	174, 177
Lactate	15.60	261, 264
Succinate	24.17	289, 293
Fumarate	25.34	287, 291
α-Ketoglutarate	31.25	346, 351
Malate	33.80	419, 423
Glutamate	37.88	432, 437
cis-Aconitate	40.63	459, 465
Glutamine	41.37	431, 436
Citrate	45.58	591, 597
Isocitrate	45.80	591, 597

Table S3. Demographic, Clinical, and Laboratory Variables of Included Patients at Admission

Parameters	FDP(n=14)
Age (years)	47.00±6.36
Males	17(89.5%)
Weight(kg)	66.69±10.29
RBC (×10^3/mm^3)	3.03 (1.98-4.61)
WBC (×10^3/mm^3)	5.59 (2.50-16.20)
Platelet (×10^3/mm^3)	104.22 (16.00-227.00)
ALT(U/L)	144.57 (16.00-1616.90)
AST(U/L)	168.47 (30.00-1023.00)
Creatinine (mg/dL)	0.66(0.37-1.35)
Bilirubin (mg/dL)	21.35 (5.67-40.77)
INR	2.09 (1.23-4.54)
Albumin (gm/dL)	36.76 (23.80-45.30)
Ammonia(umol/l)	87.42(15.7-196.6)
MELD Score	19.27±5.95
Survival	9(47.4%)[a, b]

All values are expressed as mean±SD, median (Inter-quartile range) or number (%).
[a] consistent with the currently mortality of LF as high as 50% [4, 5].
[b] no significance between FDP (fructose diphosphate) group and control groups (SMZ group (p=0.232) and LOLA group (p=0.212), respectively) from our randomized clinical trial.

FDP: fructose diphosphate; ALT, alanine transaminase; AST, aspartate transaminase; INR, international normalized ratio; MELD, model for end-stage liver disease; RBC, red blood cell; WBC, white blood cell.

Supplementary Figures and legends

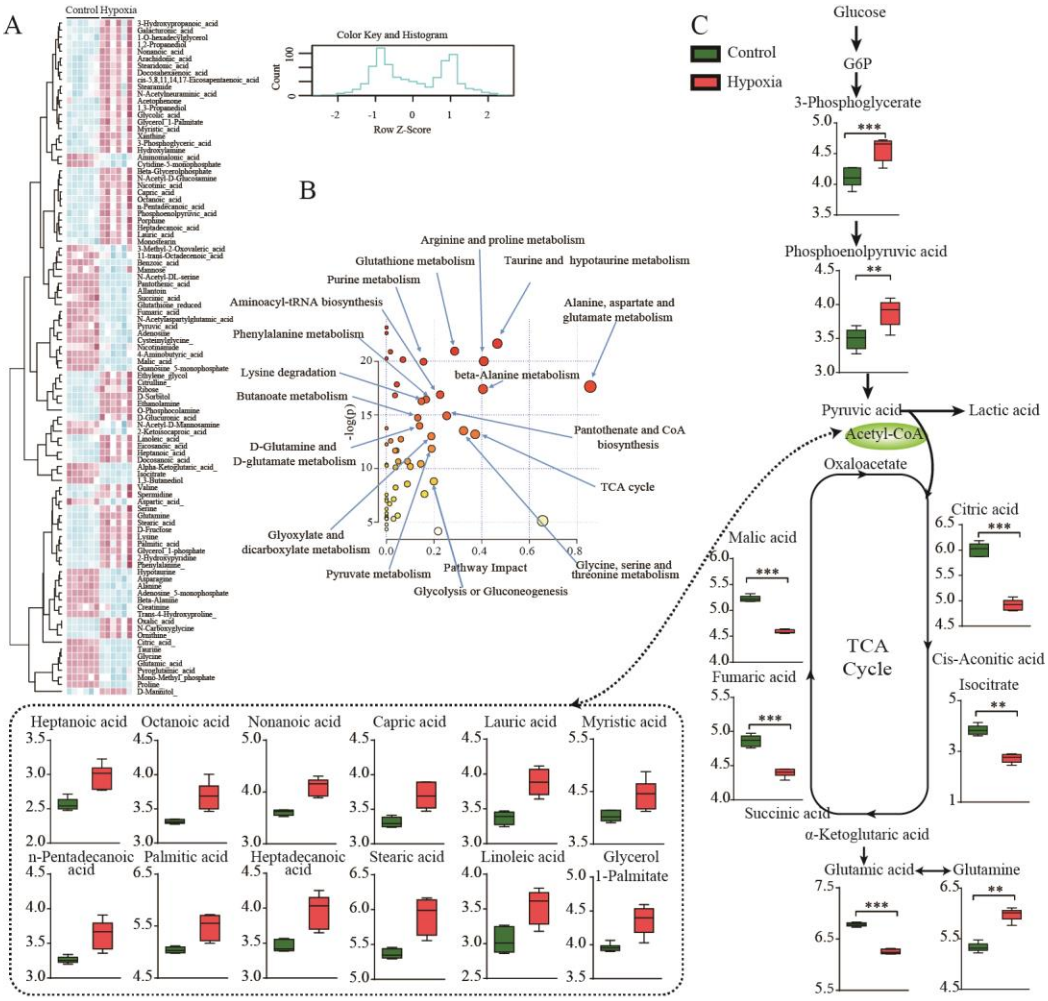

Figure S1. The metabolic status of the Chang liver cells exposed to hypoxia. Metabolomics analyses of the Chang liver cells exposed to hypoxia were performed using GC-MS and LC-MS. (A) 96 metabolites were altered after exposure of the cells to hypoxia. (B) 57 metabolic pathways were altered after exposure of the cells to hypoxia. (C) Metabolites changes of glycolysis, oxidative phosphorylation, FAO and anaplerosis of glutamine in the Chang liver cells exposed to hypoxia.

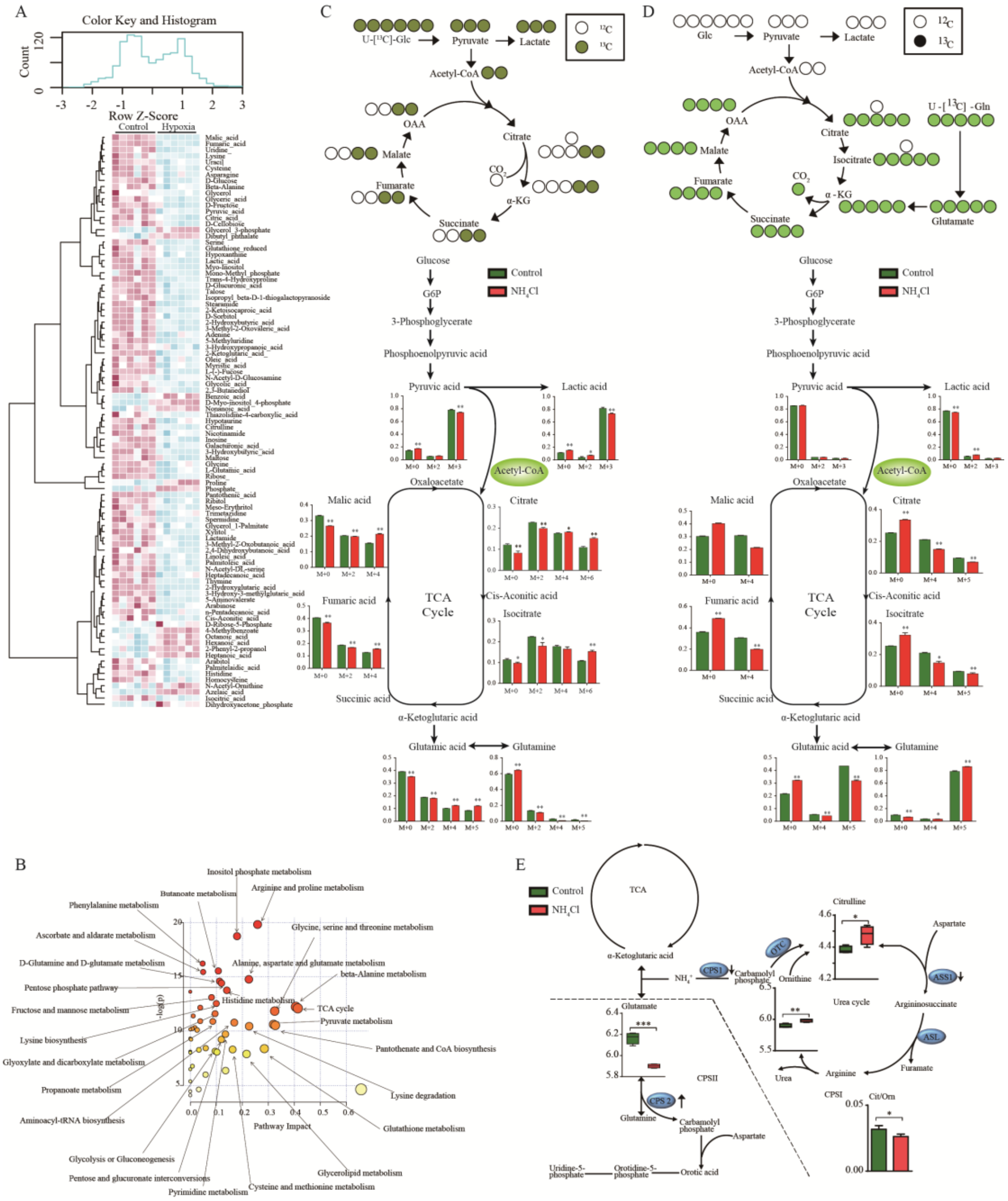

Figure S2. The metabolic status of the Chang liver cells exposed to hyperammonemia. Metabolomics analyses of the Chang liver cells exposed to hyperammonemia were performed using GC-MS and LC-MS (A) 92 metabolites were altered after exposure of the cells to hypoxia. (B) 51 metabolic pathways were altered after exposure of the cells to hypoxia. (C) The levels of glycolysis and oxidative phosphorylation were determined by stable isotope labeling techniques using ^{13}C-full labeled glucose. (D) The level of anaplerosis of glutamine was determined by stable isotope labeling techniques using ^{13}C-full labeled glutamine. (D) The level of urea cycle was determined by the ratio of Cit/Orn.

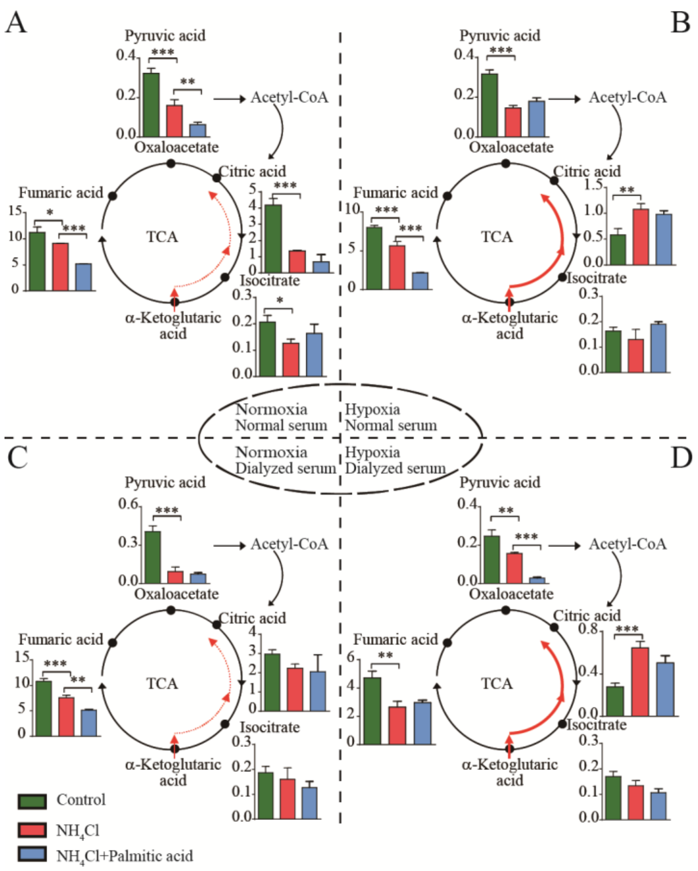

Figure S3. The effect of FAO on glycolysis in the Chang liver cells exposed to hyperammonemia was investigated using normal serum or dialyzed serum and palmitic acid. (A) The addition of palmitic acid in the culture with normal serum and normoxia. (B) The addition of palmitic acid in the culture with normal serum and hypoxia. (C) The addition of palmitic acid in the culture with dialyzed serum and normoxia. (D) The addition of palmitic acid in the culture with dialyzed serum and hypoxia.
